# Supplementary material for: Full vaccination coverage and associated factors among children aged 12 to 23 months in remote rural area of Demba Gofa District, Southern Ethiopia
Source: PeerJ. 2022 Mar 14;10:e13081. doi: 10.7717/peerj.13081 (PMC8929168; doi:10.7717/peerj.13081)
Supplement: Supplemental Information 2 [file peerj-10-13081-s002.docx]

## Annex II: English Questionnaire for Survey

Wolaita Sodo University School of Public Health

**Identification**

1. Name of District ……………………………………………………
2. Name of Kebele ……………………………………………………
3. House number given …………………………………………..

Residence: 1. Urban 2. Rural

- Result codes

| 1 = Completed  2 = Household absent  3 = Time and set for later | 4 = No competent respondent at home  5 = Incomplete interview | 6 = Refused  7 = Other: (Specify): ……… |
| --- | --- | --- |

**Part one: Socio-demographic and Economic characteristics**

|  | **Socio-demographic Questions** | | |  |
| --- | --- | --- | --- | --- |
| S.no | Questions | Answers and coding categories | Code | Skip |
| 101 | Respondent | Mother ……..…….. Care giver …………….. |  |  |
| 102 | Living condition of parents | 1=Both parents are alive 2=Mother only  3=Both parents are not alive |  |  |
| 103 | Age of mother/care giver | …………… years |  |  |
| 104 | How long did you live/stay here? | …………… months ……………. years |  |  |
| 105 | Religion | 1= Orthodox3= Muslim  2= Protestant 4= Other (specify) …………… |  |  |
| 106 | Marital status | 1= Married (in union) 2= Separated  3= Divorced4= Widowed5= Other, (specify) ………… |  |  |
| 107 | Ethnicity | 1= Gofa 2= Gamo 3= Amhara 4= Oromo 5=Gurage 6= Other, (specify) ………………… |  |  |
| 108 | Level of education of mother /care giver | ……………………….. |  |  |
| 109 | Occupation of mother /care giver | 1= House wife2= Merchant  3= Student 4= Gov’t employee  5= Daily laborer 6= Other (specify) ……………. |  |  |
| 110 | Husband’s level of education | ------------------------------ |  |  |
| 111 | Occupation of father | 1= Farmer 4= Gov’t employee  2= Merchant 5= Daily laborer  3= Student6= Other (specify) ……………… |  |  |
| 112 | Sex of child | 1= Male 2= Female |  |  |
| 113 | Number of 12-23 months old children in the family | Male= …………  Female= ………Total= …………….. |  |  |
| 114 | Birth order of last child | ………………………… |  |  |
|  | **Socio-economic (Wealth Status) Identification Questions** | | Code | Skip |
| 115 | 1. Is this your own house? 2. Number of people live in your household: 3. Does your household have the following items? 4. Does the Household have the following animals? 5. Main material of the floor 6. Main material of the roof 7. Main material of the walls 8. What type of fuel does your household use for lighting? 9. What is the main source of drinking water for members of your household? 10. What kind of latrine facility does the members of your household use? 11. Do youhaveseparateroomusedasakitchen? | 1, yes 2, no  ………………………………   \| Items \| Yes (√) \| No (√) \| Quantity \| \| --- \| --- \| --- \| --- \| \| Chair \|  \|  \|  \| \| Table \|  \|  \|  \| \| Bed \|  \|  \|  \| \| Mattress \|  \|  \|  \| \| Bicycle \|  \|  \|  \| \| Motorcy le \|  \|  \|  \| \| Telephon /mobile \|  \|  \|  \| \| Television \|  \|  \|  \| \| Radio \|  \|  \|  \| \| Farm land \|  \|  \|  \|  \| Items \| Yes (√) \| No (√) \| Quantity \| \| --- \| --- \| --- \| --- \| \| Cow /ox \|  \|  \|  \| \| Donkey/horse/mule \|  \|  \|  \| \| Goat/sheep \|  \|  \|  \| \| Chicken \|  \|  \|  \| \| Beehive \|  \|  \|  \|  1. Earth/sand 2. Wood planks 3. Cement 2. Grass 2. Corrugated iron sheet 3. Wood 2. Stone 4. Electricity 3. Charcoal 5. Wood 4. Other (specify) ………… 6. Pipe 4. Spring 7. River5. Rain water 8. Well 6. Other (specify) ………… 9. Flush latrine 3. No toilet 10. Pit latrine 4. Other (specify) …………. 11. Yes 2. No |  |  |
| 116 | Average monthly income | …………………….. ET. Birr |  |  |

**Part two: Child Vaccination characteristics**

| S.no | Questions | Answers and coding categories | Code | Skip |
| --- | --- | --- | --- | --- |
| 201 | Birth date of child | Day Month Year  …….. / ………… / ……………  Age in months …………… |  |  |
| 202 | Do your child vaccinated? | 1= Yes  2= No |  | If no, skip to Q 207 |
| 203 | If yes, do you have a card where vaccinationsare written down? | 1= Yes  2= No |  | If no, skip to Q 205 |
| 204 | If yes, copy vaccination date, month and year for each | Date Month Year  1= BCG ……… …… ………  2= Polio 0 ……… …… ………  3= Polio 1 ……… ……… ………  4= Polio 2 ……… ……… ……  5= Polio 3 ………. ……… ……  6= Penta 1 ………. ………. ……  7= Penta 2 ………. ……… ……  8= Penta 3 ………. ……… ……  9= PCV 1 ……….. ……… ……  10= PCV 2 ……….. ……… ……  11= PCV 3 ………. ……… ……  12= Rota 1 ………. ……… ……  13= Rota 2 ……….. ……… ……  14= Measles ……….. ……… ……  15= Vitamin A …….. ……… ……  16= F/vaccinated …… ……… …… |  |  |
| 205 | If no,   - - - 1. Has your child ever received BCG vaccination (an injection in the arm that usually causes a scar)? | 1. Yes  2. No  3. Don’t know |  |  |
|  | - - - 1. Has your child ever received oral polio vaccine (two drops in the mouth)?       2. If yes, how many times?       3. Has your child ever received a pentavalent vaccination (that is usually given on the left upper thigh)?       4. If yes, how many times?       5. Has your child ever received a PCV vaccination (that is usually given on the right upper thigh)?       6. If yes, how many times?       7. Has your child ever received a rotavirus vaccination (that is liquid in the mouth)?       8. If yes, how many times?       9. Has your child ever received a measles vaccination (that is an injection in the arm given at 9 months)? | 1. Yes   1. No 2. Don’t know   Number of times ……………………   1. Yes 2. No 3. Don’t know   Number of times ……………………   1. Yes 2. No 3. Don’t know   Number of times ……………………   1. Yes 2. No 3. Don’t know   Number of times ……………………   1. Yes 2. No 3. Don’t know |  |  |
| 206 | Is this child fully vaccinated according to the above information? | 1. Yes 2. No |  |  |
| 207 | If not fully vaccinate what is the child’s category? | 1. Partially vaccinated 2. Not vaccinated at all |  |  |
| 208 | If the child is defaulter, what are the reasons? | - - - 1. Vaccination site is far away       2. Vaccination time is inconvenient       3. Absenteeism of vaccinators       4. Lack of awareness       5. Not knowing the vaccination date       6. Other, (specify) ………………… |  |  |
| 209 | If the child is not vaccinated at all, what are the reasons? | 1. Absence of health facility 2. Health care provider didn’t come to give vaccination 3. Vaccination is of no use 4. Vaccination hurts children 5. Religion and culture refuses vaccination 6. Lack of awareness 7. Fear of side effects 8. Other, (specify) ………………………. |  |  |

**Part three: Maternal health care service utilization**

| S.no | Questions | Answers and coding categories | Code | Skip |
| --- | --- | --- | --- | --- |
| 301 | Maternal healthcare decision-making | 1= By herself 3= Husband alone  2= Jointly with husband 4= By others |  |  |
| 302 | Have you received ANC during your last pregnancy? | 1= Yes 2= No |  | If no, skip to Q 304 |
| 303 | If yes, how many times did you receive? | Number of times …………………… |  |  |
| 304 | Have you received TT vaccination during your last pregnancy?? | 1= Yes 2= No |  | If no, skip to Q 306 |
| 305 | If yes, how many times did you get TT injection? | Times ……………………………….. |  |  |
| 306 | Where did you give birth to your last baby? | 1= Home  2= Health institution (HC/ Hospital)  3= Other (specify) ……………………… |  |  |
| 307 | Have you attended PNC? | 1= Yes 2= No |  | If no skip to Part 4 |
| 308 | If yes, how many times? | Times ……………………….. |  |  |

**Part four: Questions on knowledge and attitude on vaccination**

| S.no | Questions | Answers and coding categories | Code | Skip |
| --- | --- | --- | --- | --- |
| 401 | Do you heard about vaccination and VPDs? | 1. Yes 2. No |  | If no, skip to Q 403 |
| 402 | If yes, from where did you hear? (Source of information) | 1. Radio 4. Friends/peers/neighbors’ 2. Television 5. Health care providers 3. From school 6. Other, (specify) ………… |  |  |
| 403 | Objective of vaccinating a child? | 1. To prevent a disease 2. For child health 3. Do not know |  |  |
| 404 | Name of VPDs mentioned by respondents | 1. Measles 3. Polio 5. Others 2. Tetanus 4. TB 6. Do not know |  |  |
| 405 | Number of VPDs known by the respondent | 1= Single disease 2= More than one disease  3= Do not know |  |  |
| 406 | Do you tell me the age at which a child begins vaccination? | 1. Just after birth 2. One month after birth 3. Do not know |  |  |
| 407 | Sessions needed to complete vaccination | 1= Three & less 3= Six & above  2= Four or five 4= Do not know |  |  |
| 408 | Age of child to complete its vaccination program? | 1= Before one year 3= Do not know  2= one year & above |  |  |
| 409 | Do you think vaccination will not make your child sick? | 1= Yes 2= No |  |  |
| 410 | Vaccinating child completely is better than partiallyvaccinated one | 1= Yes 2= No 3= Do not know |  |  |
| 411 | It is necessary to vaccinate a child who is breast feeding | 1= Strongly disagree2= Disagree  3= Neutral 4= Agree 5= Strongly agree |  |  |
| 412 | Child took usually too many vaccines | 1= Strongly disagree 2= Disagree  3= Neutral 4= Agree 5= Strongly agree |  |  |
| 413 | Vaccinations given on their own schedule | 1= Strongly disagree 2= Disagree  3= Neutral 4= Agree 5= Strongly agree |  |  |
| 414 | Vaccination prevent diseases | 1= Strongly disagree 2= Disagree  3= Neutral 4= Agree 5= Strongly agree |  |  |
| 415 | Vaccination is important for infants | 1= Strongly disagree 2= Disagree  3= Neutral 4= Agree 5= Strongly agree |  |  |
| 416 | Vaccination side effects are dangerous | 1= Strongly disagree 2= Disagree  3= Neutral 4= Agree 5= Strongly agree |  |  |
| 417 | Vaccination have no use | 1= Strongly disagree 2= Disagree  3= Neutral 4= Agree 5= Strongly agree |  |  |
| 418 | It is important to vaccinate a child who is sick | 1= Strongly disagree 2= Disagree  3= Neutral 4= Agree 5= Strongly agree |  |  |
| 419 | Vaccination make infants for death | 1= Strongly disagree 2= Disagree  3= Neutral 4= Agree 5= Strongly agree |  |  |
| 420 | If your child is not vaccinated fully, he/she can suffer to disease | 1= Strongly disagree 2= Disagree  3= Neutral 4= Agree 5= Strongly agree |  |  |

**Part five: Access to vaccination service**

| S.no | Questions | Answers and coding categories | Code | Skip |
| --- | --- | --- | --- | --- |
| 501 | Is there any health facility which gives you vaccination service near to you? | 1= Yes 2= No |  | If no skip to Q 504 |
| 502 | If yes, what kind of health facility? | 1= Health post 3= Hospital  2= Health center 4= No health facility |  |  |
| 503 | What kind of vaccination delivery strategy done EPI serving facility to you? | 1= Out reach  2= Static  3= Home to home |  |  |

Name of interviewer: ………………… Name of interviewer: …………………………………

Signature …………………………… Signature ………………………………….

Date ………………………………. Date ………………………………..
